# Supplementary material for: Model-based whole-brain perturbational landscape of neurodegenerative diseases
Source: eLife. 2023 Mar 30;12:e83970. doi: 10.7554/eLife.83970 (PMC10063230; doi:10.7554/eLife.83970)
Supplement: Supplementary file 2. — (a) Gray matter atrophy areas of participants with AD (p<0.001, FWE-cluster-corrected for multiple comparisons). (b) Gray matter atrophy areas of participants with FTD (p<0.001, FWE-cluster-corrected for multiple comparisons). [file elife-83970-supp2.docx]

**Supplementary Material: “Model-based whole-brain perturbational landscape of neurodegenerative diseases”**

Yonatan Sanz Perl^1,2,3,4*^, Sol Fittipaldi^2,3^, Cecilia González Campo^2,3^, Sebastián Moguilner^5,6^ Josephine Cruzat^4,6^, Matias E. Fraile-Vazquez^3^, Rubén Herzog^6^, Morten L. Kringelbach^7,8,9,10^, Gustavo Deco^4,11,12,13,14^, Pavel Prado^6^, Agustín Ibañez^2,3,5,6,15^, Enzo Tagliazucchi^1,2,3,6^

^1^Department of Physics, University of Buenos Aires, Buenos Aires 1428, Argentina

^2^National Scientific and Technical Research Council (CONICET), CABA, Buenos Aires, 1425, Argentina

^3^Cognitive Neuroscience Center (CNC), Universidad de San Andrés, Buenos Aires, C1011ACC, Argentina

^4^Center for Brain and Cognition, Computational Neuroscience Group, Universitat Pompeu Fabra, Barcelona, 08018; Spain

^5^Global Brain Health Institute, University of California, San Francisco, CA94158, US

^6^Latin American Brain Health Institute (BrainLat), Universidad Adolfo Ibáñez, Santiago, 8320000, Chile

^7^Department of Psychiatry, University of Oxford, Oxford, 2JD, United Kingdom

^8^Center for Music in the Brain, Department of Clinical Medicine, Aarhus University, Århus, 8000, Denmark

^9^Life and Health Sciences Research Institute (ICVS), School of Medicine, University of Minho, 4710-057 Braga, Portugal

^10^Centre for Eudaimonia and Human Flourishing, University of Oxford, Oxford, ; Oxford OX1 3JA, United Kingdom

^11^Department of Information and Communication Technologies, Universitat Pompeu Fabra, Barcelona, 08018,Spain

^12^Institució Catalana de la Recerca i Estudis Avancats (ICREA), Barcelona, 08010, Spain

^13^Department of Neuropsychology, Max Planck Institute for Human Cognitive and Brain Sciences, Leipzig, 04103, Germany

^14^School of Psychological Sciences, Monash University, Melbourne, Clayton VIC 3800, Australia

^15^Trinity College Institute of Neuroscience (TCIN), Trinity College Dublin, Dublin, D02DP21, Ireland

* Corresponding author: [yonatan.sanz@upf.edu](mailto:yonatan.sanz@upf.edu)


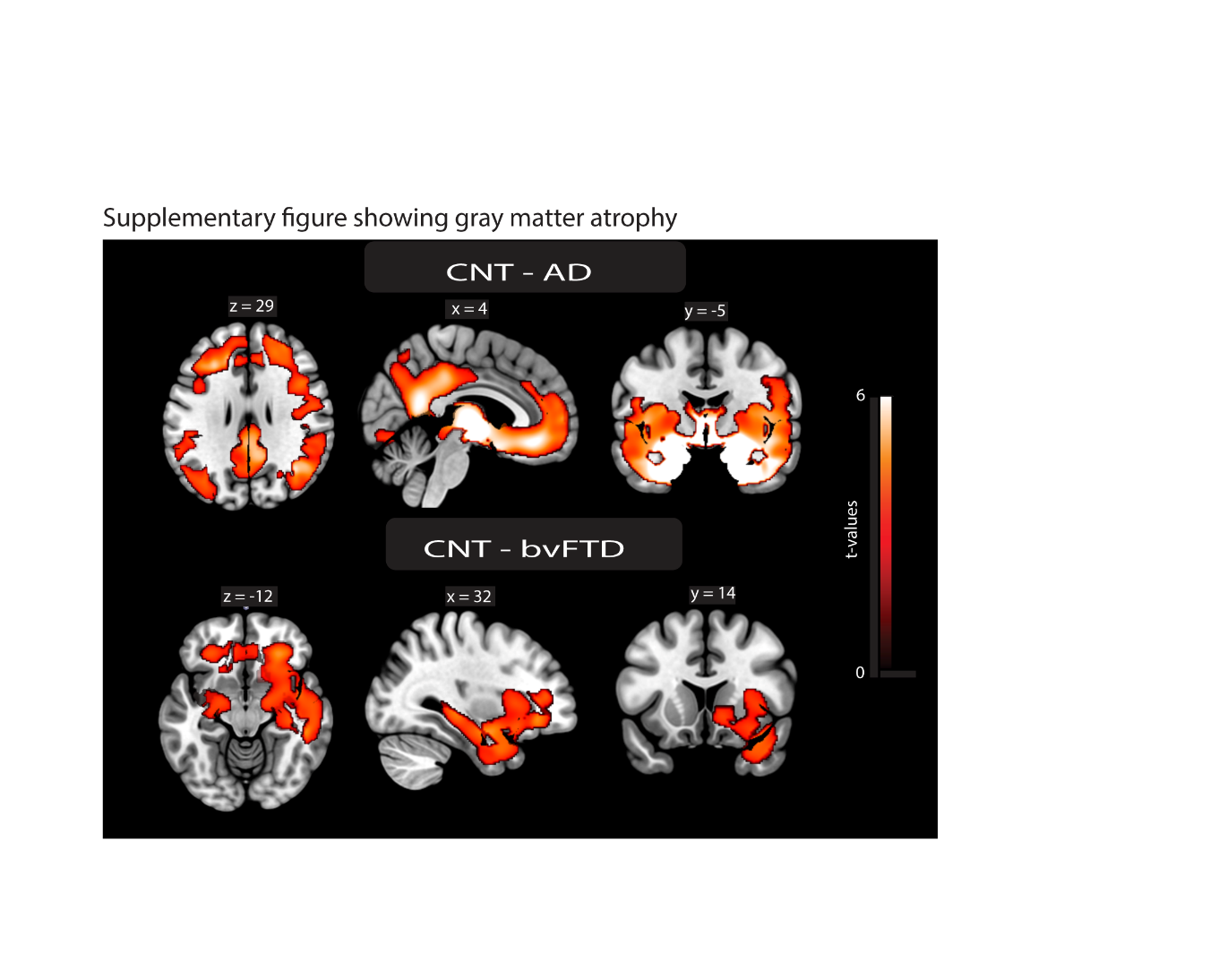


***Figure 2 — figure supplement 1:*** ***atrophy maps****.* ***Voxel-based morphometry analysis.*** *The atrophy pattern of participants with AD and bvFTD was calculated by comparing their grey matter W-maps with those of controls using two-sample t-tests in SPM12. The alpha level was set at p < .001, cluster-corrected for multiple comparisons. Localization was derived from the AAL atlas.* *The brain renders of atrophy maps of AD (upper panel) and bvFTD (lower panel) compared to CNT.*

| Number of voxels | *t*-value | *p*-value | MNI coordinates | | | Brain area (AAL) |
| --- | --- | --- | --- | --- | --- | --- |
|  |  |  | x | y | z |  |
| 160672 | 10,6 | <.001 | -30 | -12 | -15 | Hippocampus L |
|  | 10,3 | <.002 | -19,5 | -6 | -18 | Amygdala L |
|  | 10,1 | <.003 | 30 | -10,5 | -16,5 | Hippocampus R |

***Supplementary file 2a:***  Gray matter atrophy areas of participants with AD (p < .001, FWE-cluster-corrected for multiple comparisons).

| Number of voxels | *t*-value | *p*-value | MNI coordinates | | | Brain area (AAL) |
| --- | --- | --- | --- | --- | --- | --- |
|  |  |  | x | y | z |  |
| 18409 | 5,0 | <.001 | 49,5 | -13,5 | -21 | Temporal Lobe R |
|  | 4,9 | <.001 | 43,5 | 6 | -27 | Mid Temporal Pole R |
|  | 4,8 | <.001 | 46,5 | -4,5 | -27 | Mid Temporal Lobe R |
| 1341 | 4,4 | <.001 | -36 | 19,5 | 16,5 | Inf Frontal L |
|  | 4,0 | <.001 | -34,5 | 15 | 6 | Insula L |
|  | 3,4 | <.001 | -24 | 21 | -1,5 | Frontal Lobe L |
| 2959 | 4,2 | <.001 | -31,5 | -4,5 | -18 | Parahippocampa Gyrus L |
|  | 3,7 | <.001 | -40,5 | 0 | -30 | Mid Temporal L |

***Supplementary file 2b:***  Gray matter atrophy areas of participants with bvFTD (p < .001, FWE-cluster-corrected for multiple comparisons).


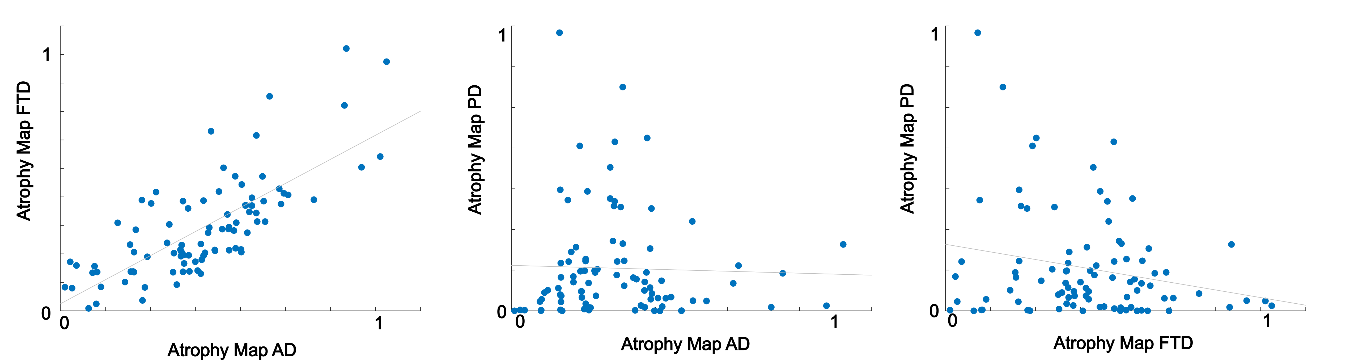


***Figure 2 — figure supplement 2: Correlations between disease-atrophy maps****. The correlation between the atrophy map of AD and FTD is high (R=0.75,p<0.001), left panel; while the correlations between the FTD and AD maps and the PD maps are lower (PD vs Ad; R=-0.03,p=0.76; PD vs FTD; R=-0.22, p=0.05).*


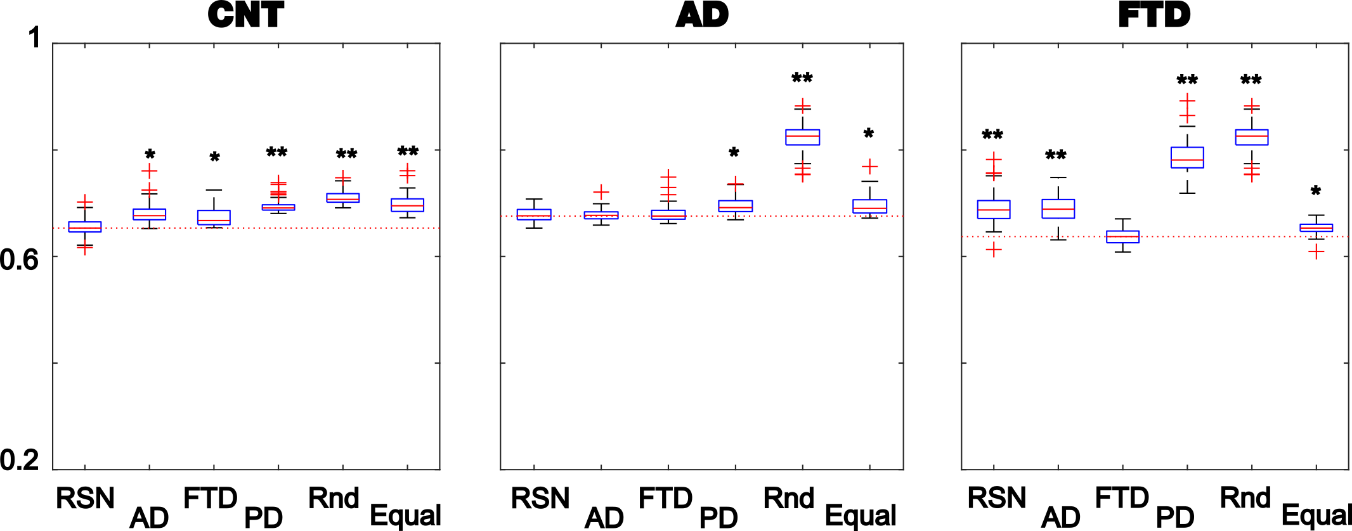


***Figure 2 — figure supplement 3:*** ***Fitting the whole-brain model to the empirical data****. The violin plots display 1 - GoF values (300 independent realizations of parameter fitting) for CNT, AD and bvFTD using anatomical priors based on resting state networks (RSN), AD, bvFTD (separately), and Parkinson’s disease atrophy maps (PD), random assignment (Random), and equally sized groups of nodes defined by anatomical proximity (Equal) (* and ** indicate large [*$\left| d \right|$*>0.8] and very large [*$\left| d \right|$*>1.3] effect sizes according to Cohen’s d computed against the best fitting prior of each model)*


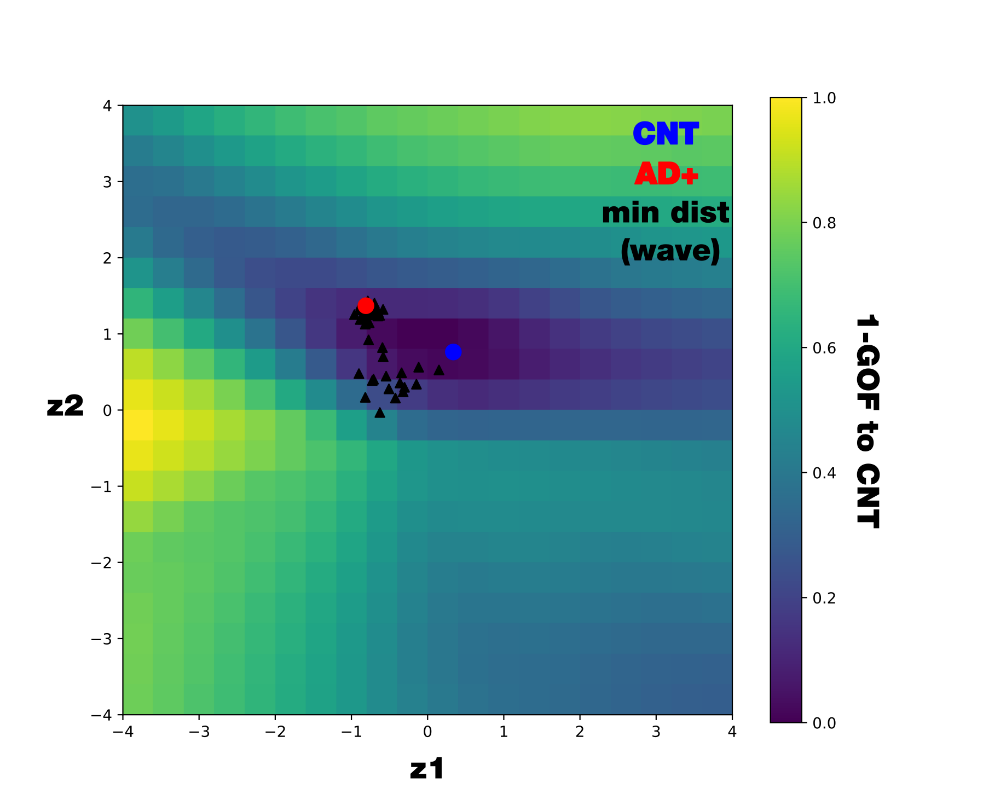


***Figure 5 – figure supplement 1:***  ***Mapping 1-GOF in the latent space comparing with distance to CNT.*** We *systematically decoded points within a 20x20 grid in the latent space and compute the GOF between the decoded FCs and the controls’ FC. We display the GOF map together with the controls’ centre (blue circle), AD+ centre (red circle) and all the minimal distance of all the possible perturbations of the AD+ condition (in the wave perturbative approach, black triangles). As is noticeable, not equal distances representing equal GOF this is due to the two-dimensional nature of the latent space representation.*
